# The Effect of Limbal Fenestrations on Scleral Lens-Induced Central Corneal Oedema

**DOI:** 10.1007/s44402-026-00052-0

**Published:** 2026-03-09

**Authors:** Damien Fisher, David Alonso-Caneiro, Stephen J. Vincent

**Affiliations:** 1 Centre for Vision and Eye Research, Optometry and Vision Science, Queensland University of Technology, Brisbane, Australia; 2 School of Science, Technology and Engineering, University of the Sunshine Coast, Petrie, Australia

**Keywords:** Corneal oedema, Fenestration, Oxygen, Scleral lens

## Abstract

**Purpose:**

Persistent corneal oedema can lead to reduced visual performance and longer-term ocular complications in eyes with reduced endothelial cell function. The aim of this study was to quantify the effect of incorporating limbal scleral lens fenestrations on central corneal oedema.

**Methods:**

Twenty young healthy adults (mean age ± standard deviation 32 ± 11 years) with normal corneas wore non-fenestrated and fenestrated (3 × 1 mm limbal fenestrations separated by 120°) OneFitMed+ scleral lenses in both eyes (on separate days) for 3 h. All other scleral lens parameters were held constant except for the addition of the fenestrations. Central fluid reservoir thickness and corneal oedema were quantified using optical coherence tomography, and corrections were made for small thickness variations between lens conditions.

**Results:**

The corrected central corneal oedema was significantly reduced following fenestrated lens wear (0.80 ± 0.93%) compared to non-fenestrated lens wear (1.32 ± 0.81%; a 39% relative reduction, *p* = 0.02). The reduction in central corneal oedema with the fenestrated lens was slightly greater for participants without an air bubble in the fluid reservoir (*n* = 10, 0.73 ± 0.80% less oedema, 45% relative reduction) compared to those with an air bubble (*n* = 10, 0.30 ± 1.05% less oedema, 30% relative reduction) but was not statistically significant (*p* = 0.31).

**Conclusion:**

The incorporation of three 1 mm diameter limbal fenestrations separated by 120° reduced the magnitude of central corneal oedema by 39% on average in healthy eyes compared to a non-fenestrated scleral lens. A reduction in central corneal oedema was still observed without the presence of an air bubble within the fluid reservoir. Future studies investigating the long-term efficacy of different fenestration configurations in clinical populations are warranted.

**Clinical trial:**

This study was registered on the Australian New Zealand Clinical Trials Registry (Registration number 12622001164785p), date of registration 24 August, 2022.

Key Points

- When controlling for fluid reservoir thickness and lens oxygen transmissibility, three 1 mm diameter limbal fenestrations separated by 120° reduced scleral lens-induced central corneal oedema by 39%.
- Fenestrated scleral lenses, both with or without a fluid reservoir bubble, reduced central corneal oedema, suggesting that oxygen or carbon dioxide transfer may be altered with fenestrations.
- In comparison to a single limbal fenestration, a greater number of fenestrations or a larger fenestrated area, results in less scleral lens-induced central corneal oedema.

## Introduction

In healthy adults with normal endothelial function, highly oxygen-permeable scleral lenses typically induce ~2% central corneal oedema during open eye wear [1–3]. However, in patients with a corneal graft and reduced endothelial cell function, persistent oedema remains a significant complication [4–9] that can lead to neovascularisation and potentially graft rejection or failure in the long term. Scleral lenses are often used to manage high degrees of corneal astigmatism following keratoplasty, accounting for ~17% of scleral lens wearers [10].

In addition to corneal endothelial function, modifiable factors which contribute to corneal oedema in scleral lens wear include the oxygen permeability [11, 12] and thickness [13–16] of the lens and fluid reservoir [17, 18] and the extent of tear exchange [19, 20]. Tear exchange during contact lens wear facilitates the removal of oxygen-depleted tears from behind the lens that contain corneal metabolic waste products and tear film debris (e.g., lipid, mucous, environmental contaminants) [21], and replenishes the cornea with fresh, oxygen-rich tears [22, 23].

A number of different approaches have been used in clinical practice in an attempt to enhance tear exchange during scleral lens wear, including fitting slightly flat in the landing zone, or incorporating lens modifications such as back surface channels or fenestrations [24]. Although fenestrations have been utilised since the glass scleral lenses of the 1940’s, few studies have quantified the effect of incorporating lens fenestrations on corneal oedema. In 1970, Ko et al. [25] examined tear exchange in scleral lenses containing a single limbal fenestration and reported variable results in a limited sample of four eyes. The authors concluded that adding a fenestration may not improve corneal oxygenation; however, oedema was not measured. More recently, Fisher et al. [26] quantified the magnitude of central corneal oedema following a short period of fenestrated (one 0.3 mm limbal fenestration) and non-fenestrated scleral lens wear. No statistically significant difference in oedema was observed between the two lens designs (mean of 0.62% oedema for non-fenestrated and 0.50% for fenestrated). However, this difference equates to a 19% relative reduction in corneal oedema, which may be clinically relevant when the overall magnitude of oedema is substantially greater (e.g., due to compromised corneal endothelial cell integrity or overnight lens wear for corneal rehabilitation).

Several fenestration configurations have been trialled for scleral lenses, but a common approach includes three fenestrations, each separated meridionally by 120°, to ensure at least one is always located within the palpebral aperture if the lens rotates [27], with fenestration sizes varying from 0.1 to 2 mm. Numerous studies [28–30] have advocated the benefits of fenestrated scleral lenses in clinical populations such as longer comfortable wearing time, delay in the onset of visual symptoms (such as Sattler’s veil), which have been attributed to improved tear exchange and corneal oxygenation. However, to date, studies quantifying the effect of different configurations of fenestrations have been limited to corneal rigid [31, 32] or soft contact lenses [33]. Therefore, the aim of this study was to quantify the effect of incorporating three equally spaced 1 mm diameter limbal fenestrations into a highly oxygen-permeable scleral lens on central corneal oedema.

## Methods

The study was approved by the Queensland University of Technology Human Research Ethics Committee and conducted in accordance with the tenets of the Declaration of Helsinki. The study was registered as a clinical trial with the Therapeutics Goods Administration and the Australian and New Zealand Clinical Trials Registry (ACTRN12622001164785p), date of registration 24 August, 2022. All participants provided informed consent to participate.

### Visit 1: Preliminary Screening

An initial ophthalmic screening examination was conducted to ensure the study inclusion criteria were met. This included an ocular and medical history, measurement of visual acuity in each eye, and an anterior ocular health assessment to ensure suitability for contact lens wear. All participants had visual acuity of 0.00 logMAR or better in each eye, with no contraindications to contact lens wear, and no history or evidence of any ocular disease, injury or surgery. Rigid contact lens wearers were excluded from participating. Soft contact lens wearers were included, but ceased lens wear for 24 h prior to each study visit.

### Visit 1: Scleral Lens Fitting

The corneal sagittal depth across a 10-mm chord along the steep corneal meridian was measured using a corneal topographer (E300, Medmont, medmont.com.au) and extrapolated to a 15-mm chord by adding 2000 µm to this value. An additional 275 µm was then added to this value to determine the sagittal depth of the first scleral lens to trial on the eye (aiming for an initial central fluid reservoir thickness of ~250 µm). Fenestrated and non-fenestrated scleral lenses were then fitted to both right and left eyes with preservative-free saline (Lens Plus Ocupure, Abbott Medical Optics, abbott.com) used as the application fluid to fill the scleral bowl. The central fluid reservoir thickness was quantified using an optical coherence tomographer (OCT) (Spectralis, Heidelberg Engineering, heidelbergengineering.com) and the fit of the lens was assessed using a slit lamp biomicroscope. If the initial lens selected resulted in insufficient or excessive central corneal clearance, a different lens with a different sagittal depth was applied and the fit was reassessed. Lenses were then worn for approximately 30 min to ensure there was no discomfort or ingress of a central air bubble within the fluid reservoir.

**Fig. 1:**
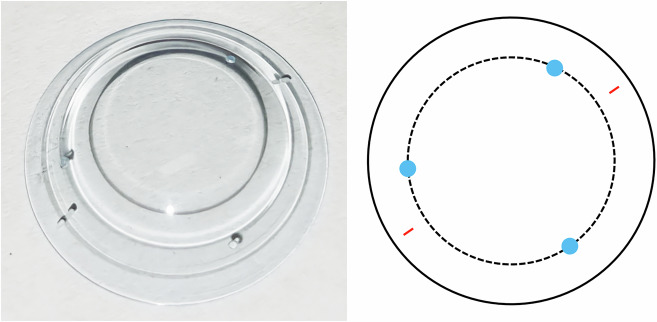
Image of a 17-mm total diameter fenestrated lens used in the study (left) and corresponding schematic overview drawn to scale (right). Three 1 mm fenestrations (blue dots) were positioned 120° apart, 6 mm from the lens centre (dashed line). Red lines are the toric line markers.

Non-fenestrated scleral lenses (OneFitMed + , No. 7 Contact Lenses, no7contactlenses.com) manufactured in roflufocon A material (Dk 100) were used with the following parameters: sagittal depths ranging from 4600 to 5200 µm in 100 µm increments, 17 mm total diameter, −1.00 D back vertex power, standard prolate back surface design and 200 µm landing zone toricity. Fenestrated lenses were manufactured using the same parameters but with the inclusion of three 1 mm diameter fenestrations positioned 6 mm from the lens centre at the 4, 8 and 12 o’clock locations (i.e., separated by 120°) (Fig. 1). The thickness profile of the fenestrated and non-fenestrated lenses used in the study were determined across the central 13 mm (the approximate region covering the cornea) using an OCT imaging technique [34]. This technique calculates the lens thickness along the likely path of oxygen flow through the material.

### Visits 2 and 3: Fenestrated and Non-Fenestrated Lens Wear

During Visits 2 and 3, participants wore the optimal fitting scleral lenses (determined at Visit 1) in both eyes (e.g., Visit 2 non-fenestrated lenses in both eyes, Visit 3 fenestrated lenses in both eyes) for 3 h. To minimise the effect of any potential systematic bias introduced by the order of lens wear across the study visits, the order of lens wear (fenestrated/non-fenestrated lenses) was counter-balanced across participants. Visits 2 and 3 were separated by at least 24 h and scheduled for the same time of day for each individual participant (at least 2 h after waking) to minimise the influence of diurnal variations in corneal thickness [35].

The lens was applied to the right eye with preservative-free saline (Lens Plus Ocupure, Abbott Medical Optics, abbott.com) as the application fluid (and the lens toric line markers aligned along the 0–180 meridian) and baseline images were obtained with the OCT. The same procedure was then carried out for the left eye. OCT imaging was repeated after 180 min of lens wear since scleral lens induced central corneal oedema peaks and stabilises after approximately 90 min in healthy eyes [1]. The OCT imaging protocol was a high-resolution volumetric scan centred on the pupil consisting of three horizontal line scans separated by 139 µm, each with 20 averaged B-scans in each line scan. The OCT used in this experiment (Spectralis, Heidelberg Engineering, heidelbergengineering.com) has an axial resolution of 3.9 µm. Three volumetric scans were taken at each time point and exported for further analysis. All images were analysed by a single experienced observer. This technique is highly repeatable with a mean intrasession repeatability of less than 1 µm for measures of total corneal thickness (when averaging three line scans) [36].

### Image Analysis

The OCT data were analysed using customised software which allows the user to segment the contact lens and corneal boundaries of interest. OCT analysis was undertaken by a single experienced examiner who was not masked to the lens wear condition. Corneal thickness data were averaged across the central 6 mm, and the fluid reservoir thickness was determined at the corneal apex in the cross-sectional OCT images. The OCT data presented is the average of three scans at each time point for the right eye only to maintain statistical independence, given the high degree of interocular symmetry between fellow eyes [37].

### Statistical Analysis

To account for small differences in the fluid reservoir thickness and lens thickness between the fenestrated and non-fenestrated lens conditions (mean ≤9 µm) (Table 1), a correction factor was applied using data from previous work which quantified the effect of the lens [15] and fluid reservoir thickness [17] upon central corneal oedema during open eye scleral lens wear in young healthy adults. The mean polynomial equations from these previous studies [15, 17] were used to estimate the magnitude of corneal oedema due to a difference in lens or fluid reservoir thickness between the fenestrated and non-fenestrated conditions in the current experiment. The measured corneal oedema was then corrected by subtracting this estimated difference from the oedema value of the condition with the greater lens or fluid reservoir thickness. This approach has been used previously in a study comparing fenestrated and non-fenestrated scleral lenses [26]. Both the raw and corrected oedema data are presented.

**Table 1:** Central corneal oedema and lens and fitting characteristics for the fenestrated and non-fenestrated conditions for all participants.

Parameter	Non-fenestrated (*n* = 20)	Fenestrated (*n* = 20)	*p* value (paired *t* test)
Lens thickness (averaged across 13 mm) (µm)	276 ± 9	283 ± 8	0.03
Initial central fluid reservoir thickness (µm)	258 ± 34	249 ± 39	0.37
Final central fluid reservoir thickness (µm)	194 ± 33	179 ± 42	0.05
Central lens settling (µm)	66 ± 36	69 ± 29	0.52
Raw central oedema (6 mm) (%)	1.41 ± 0.84	0.86 ± 0.95	0.02
Corrected central oedema (6 mm) (%)	1.32 ± 0.81	0.80 ± 0.93	0.02

The study sample size was chosen based on a repeated-measures study [26] comparing scleral lens-induced corneal oedema between fenestrated and non-fenestrated lenses (*n* = 9). Twenty participants provided an effect size of 0.6 and a power of 0.7 to detect significant differences between the two conditions for a paired *t* test with a 0.05 alpha level. The normality of the data was assessed using the Kolmogorov–Smirnov test. Paired *t* tests were used to compare lens thickness, fluid reservoir thickness and corneal oedema between the fenestrated and non-fenestrated lens conditions. An unpaired *t* test was used to compare the difference in corneal oedema between the two lenses for participants with (*n* = 10) and without (*n* = 10) a fluid reservoir bubble during the fenestrated lens condition.

#### Ethics Approval and Consent to Participate

This study received ethics approval from the Queensland University of Technology Human Research Ethics Committee.

## Results

Twenty young healthy adults (mean age 32 ± 11 years, 10 females/10 males) completed the study. There was a diverse range of ethnicities, including Asian (7), Caucasian (5), Indian (4), Middle Eastern (2), African (1) and Nepalese (1). Six participants were regular soft lens wearers and ceased lens wear for at least 24 h prior to each study visit. Half the participants were fitted with a mobile peripheral bubble in the right eye during the fenestrated lens condition, which did not impact subjective vision. Twelve potential participants were excluded from participating in Visits 2 and 3 due to the ingress of a large central bubble within the fluid reservoir during the Visit 1 scleral lens fitting.

### Central Corneal Oedema

**Fig. 2:**
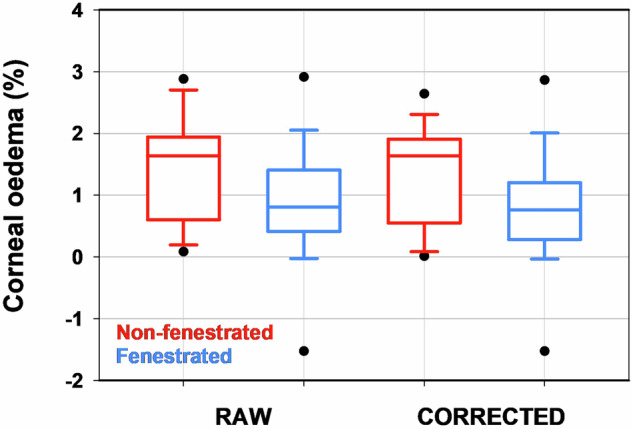
Box plots of raw and corrected corneal oedema for the non-fenestrated (red) and fenestrated (blue) lens conditions. The upper and lower boundaries of the box are the 75th and 25th percentiles, respectively, and the line within the box is the median. The upper and lower whiskers are the 90th and 10th percentiles, respectively, and the black dots represent the 95th and 5th percentiles.

The lens and fluid reservoir thickness values and the raw and corrected central corneal oedema for the non-fenestrated and fenestrated lens conditions are displayed in Table 1. On average, the incorporation of three 1 mm limbal fenestrations resulted in a 39% relative reduction in central corneal oedema after correcting for small differences in lens and fluid reservoir thickness between the lens conditions (Table 1; Fig. 2).

**Fig. 3:**
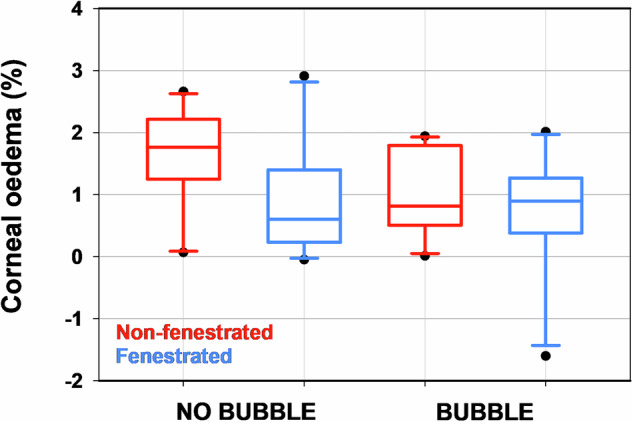
Box plots of the corrected central corneal oedema for the non-fenestrated (red) and fenestrated (blue) lens conditions for participants with (*n* = 10) and without (*n* = 10) an air bubble within the fluid reservoir for the fenestrated lens. The upper and lower boundaries of the box are the 75th and 25th percentiles, respectively, and the line within the box is the median. The upper and lower whiskers are the 90th and 10th percentiles, respectively, and the black dots represent the 95th and 5th percentiles.

The reduction in central corneal oedema with the fenestrated lens was slightly greater for participants without an air bubble in the fluid reservoir (0.73 ± 0.80% less oedema, 45% relative reduction) compared to those with an air bubble (0.30 ± 1.05% less oedema, 30% relative reduction), but this difference was not statistically significant (*p* = 0.31) (Fig. 3).

## Discussion

This is the first study to quantify the effect of three limbal 1 mm scleral lens fenestrations upon corneal oedema. After correcting for differences in lens thickness and fluid reservoir thickness, fenestrated lenses resulted in a 39% reduction in central corneal oedema compared to non-fenestrated lenses (mean oedema 1.32% for non-fenestrated and 0.80% for fenestrated). Using a similar experimental approach, Fisher et al. [26] reported that a single 0.3 mm limbal fenestration reduced central oedema by 19% compared to a non-fenestrated control condition. Together, these findings suggest that a greater number of fenestrations (or larger fenestrated area) results in less scleral lens-induced central corneal oedema.

Efron and Carney [33] investigated the effect of various soft lens fenestration configurations upon equivalent oxygen percentage (EOP) in comparison to a non-fenestrated control lens. Four 1.8-mm mid-peripheral fenestrations resulted in a significant increase in EOP beneath the lens; however, all participants reported discomfort with this design. Other configurations (4 × 0.8 mm and 8 × 0.8 mm diameter mid-peripheral fenestrations) with a lower fenestrated area did not alter oxygen availability at the cornea. Harris et al. [31] also observed a decrease in central corneal oedema with an increasing number of 0.1 mm mid-peripheral fenestrations in polymethylmethacrylate corneal lenses. Compared to a non-fenestrated lens, three fenestrations resulted in a 17% relative reduction, which increased to a 48% reduction for 20 fenestrations. In the current scleral lens study, the fenestrated lens area was 2.36 mm^2^ compared to 0.07 mm^2^ in Fisher et al. [26]. While increasing the size or number of scleral lens fenestrations may further reduce central oedema, based on clinical experience, the ingress of a large fluid reservoir bubble is more likely and can be difficult to maintain at a peripheral location away from the visual axis.

The finding that central corneal oedema was reduced with (30% reduction) or without (45% reduction) a fluid reservoir air bubble suggests that the mechanism underlying the reduction in central corneal swelling may arise due to increased oxygen ingress or carbon dioxide egress through the fenestration, or enhanced tear exchange either through the fenestration or via the landing zone (due to reduced suction forces). In early fenestration experiments with scleral lenses, Smelser et al. [38] hypothesised that Sattler’s veil was alleviated as the cornea drew in oxygen from air bubbles within the reservoir. However, Fadel and Ezekiel [27] noted that a bubble may not form with corneal alignment, and the lens is still effective.

The main limitations of the current study were the inclusion of healthy participants with normal corneas and the short duration of lens wear. Longer-term studies are required to assess the clinical efficacy of fenestrated scleral lenses in eyes prone to corneal oedema (e.g., post-keratoplasty or post-radial keratotomy). Precisely matching the lens and fluid reservoir thickness across the two lens conditions was not possible (Table 1). While the mean differences between the fenestrated and non-fenestrated lenses were less than 15 µm, a correction factor was applied using previously published data describing the change in central oedema as a function of lens and fluid reservoir thickness. Peripheral corneal oedema was not quantified in this study, but would likely be reduced relative to the central region based on the regional variations observed for a smaller diameter fenestration [39].

In conclusion, the incorporation of three 1 mm diameter limbal fenestrations separated by 120° into a highly oxygen-permeable scleral lens reduced the magnitude of central corneal oedema by 39% on average in healthy eyes compared to a non-fenestrated scleral lens, when adjusting for differences in central fluid reservoir thickness and scleral lens thickness between conditions. This reduction in central oedema was greater than previously reported for a 0.3-mm diameter fenestration (19% relative reduction) and was observed irrespective of the presence of a fluid reservoir bubble (with bubble 30%, without 45%). Future studies investigating the long-term efficacy of different fenestration configurations in clinical populations are warranted.

## Data Availability

All data supporting the findings of this study are available within the paper.
